# Chromosome-level genome assembly of the snakefly *Mongoloraphidia duomilia* (Raphidioptera: Raphidiidae)

**DOI:** 10.1038/s41597-024-03439-1

**Published:** 2024-06-04

**Authors:** Rongrong Shen, Terrence Sylvester, Na Ra Shin, Zhihong Zhan, Jianfeng Jin, Ding Yang, Duane D. McKenna, Xingyue Liu

**Affiliations:** 1 Department of Entomology, China Agricultural University, Beijing, 100193 China; 2 Department of Biological Sciences, University of Memphis, Memphis, TN 38152 USA; 3 Center for Biodiversity Research, University of Memphis, Memphis, TN 38152 USA; 4 Department of Entomology, Nanjing Agricultural University, Nanjing, 210095 China

**Keywords:** Evolution, Genetics

## Abstract

Raphidioptera (snakeflies) are a holometabolan order with the least species diversity but play a pivotal role in understanding the origin of complete metamorphosis. Here, we provide an annotated, chromosome-level reference genome assembly for an Asian endemic snakefly *Mongoloraphidia duomilia* (Yang, 1998) of the family Raphidiidae, assembled using PacBio HiFi and Hi-C data from female specimens. The resulting assembly is 653.56 Mb, of which 97.90% is anchored into 13 chromosomes. The scaffold N50 is 53.50 Mb, and BUSCO completeness is 97.80%. Repetitive elements comprise 64.31% of the genome (366.04 Mb). We identified 599 noncoding RNAs and predicted 11,141 protein-coding genes in the genome (97.70% BUSCO completeness). The new snakefly genome will facilitate comparison of genome architecture across Neuropterida and Holometabola and shed light on the ecological and evolutionary transitions between Neuropterida and Coleopterida.

## Background & Summary

Raphidioptera (snakeflies) are a relictual group of holometabolous insects whose species diversity was higher in the Mesozoic than today^1^. The extant species of Raphidioptera comprises two families, Raphidiidae and Inocelliidae, with 31 genera and 253 described extant species^2^. They are restricted to forested parts of the Holarctic^1,3^ but do not occur in northern and eastern parts of North America. Adult Raphidiidae are diurnal predators of soft-bodied arthropods, but Inocelliidae also feed on pollen. All larval stages are entomophagous and feed on various soft-bodied arthropods; however, the prey spectrum differs considerably between bark and soil-dwelling larvae. A few days before hatching of the adult, the snakefly pupa becomes very active and is able to run. Development in Raphidioptera is highly plastic and depends on environmental conditions^1,4,5^. For example, while Raphidioptera are not sensitive to changes in the photoperiod, they are highly sensitive to temperature. Raphidioptera need a period of lowered temperature (usually around 0 °C) to induce pupation or hatching^6^. Larvae continuously kept at room temperature typically will not pupate and instead become prothetelous, i.e., developing pupal characters, such as compound eyes, wing pads, and appendages on the abdomen, and may live for multiple years. While many species of Raphidioptera are uncommon, some can be locally abundant, and there have been several attempts to use snakeflies as biological control agents^7^.

Raphidioptera has been recovered as the sister group of Megaloptera plus Neuroptera based on recent morphology-based and genome-based phylogenetic analyses^3,8^. However, phylogenetic relationships among genera or species within Raphidioptera are still little known. Few studies have reconstructed the intergeneric phylogeny of Raphidiidae using multi-loci DNA sequence data^8^ and shared orthologous DNA sequences^9^, or the intergeneric phylogeny of Inocelliidae using mitochondrial genome data (Shen *et al*., 2022). Due to the lack of large-scale genomic data for Raphidioptera, the biogeographical history of their Holarctic distribution and the genetic mechanisms underlying their development and adaptation remain unresolved.

Raphidioptera is represented by only two genomes in NCBI (assessed: Nov 11, 2023). The only formally published raphidiopteran genome is from the black-necked snakefly, *Venustoraphidia nigricollis* Albarda, 1891 (assembly ID: JAVRKA000000000), and this has not been assembled to the chromosome level^9^. Notably, Neuropterida was entirely lacking from the study of gene content evolution in arthropods by Thomas *et al*., owing to a lack of suitable genomes^10^. Here we sequenced and assembled a high-quality reference genome of an Asian endemic raphidiid species *Mongoloraphidia duomilia* (Yang, 1998), representing the first snakefly genome assembled to the chromosome level. The genus *Mongoloraphidia* H. Aspöck and U. Aspöck, 1968 contains more than 60 described species, comprising approximately one-third of the world species of Raphidiidae, and *M. duomilia* is relatively widespread across several provinces of northern China^11^. The new snakefly genome will facilitate studies of ecological and evolutionary transitions in Neuropterida and its close relatives, such as Coleopterida (Coleoptera + Strepsiptera) (e.g.^12–16^, and provide a new and much needed^17,18^ point of comparison for studies of genome architecture and evolution across Neoropterida and Holometabola.

## Methods

### Sample collection and sequencing

**Fig. 1:**
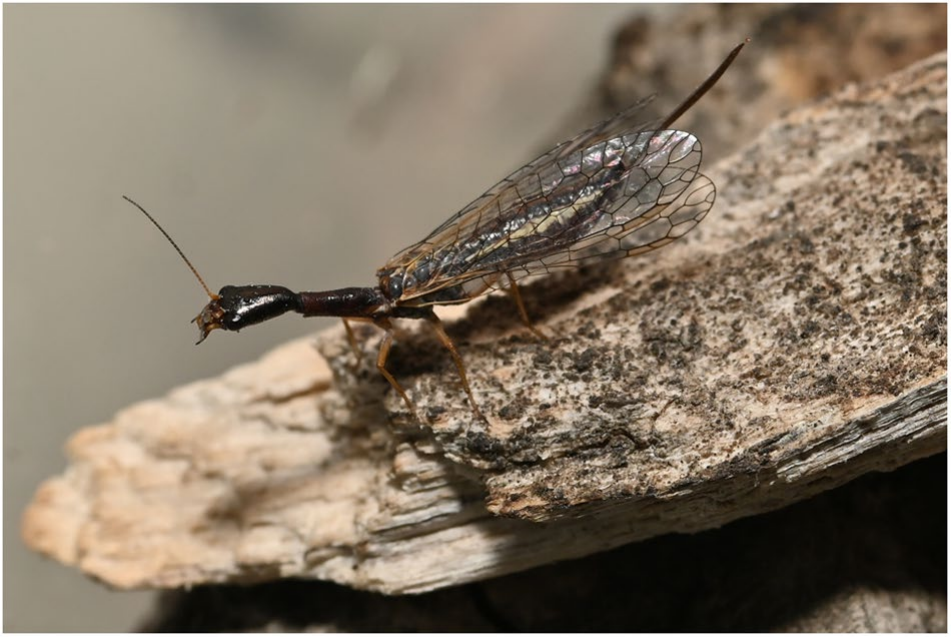
Live adult of *Mongoloraphidia duomilia*, male.

Adult specimens of *Mongoloraphidia duomilia* were collected on June 02, 2022, at Bauhausian National Nature Reserve, Mentougou District, (39°50′19″N, 115°34′22″E; 1197.9 m), Beijing, China (Fig. 1). The samples were initially placed in liquid nitrogen and stored at −80 °C before DNA extraction. After DNA extraction, the specimens were deposited in the Entomological Museum of China Agricultural University (CAU), Beijing, China.

Two female adult specimens were used for PacBio HiFi and Illumina whole genome sequencing. Additionally, two separate individual females were used for Iso-Seq and Illumina transcriptome sequencing. A single female was used for Hi-C sequencing. Genomic DNA and RNA were extracted using the Qiagen Blood & Cell Culture DNA Mini Kit and TRIzol^TM^ Reagent, respectively. Two different kinds of SMRTbell® 2.0 libraries were prepared for sequencing: (1) a PacBio single-molecule real-time (SMRT) library with a 20 kb insert size (for genome sequencing) and (2) an Iso-Seq library (for transcriptome sequencing) without size selection. Illumina libraries were prepared with a 350 bp insert size using the TruSeq DNA PCR-Free LT Library Preparation Kit and TruSeq RNA v2 Kit (Illumina, San Diego, CA, USA). The restriction enzyme DpnII was used to digest DNA for the Hi-C assay. Short-read and long-read libraries were sequenced on Illumina NovaSeq 6000 (150 bp paired-end reads) and PacBio Sequel II platforms, respectively. Finally, we obtained 41.57 Gb (64.14X) of PacBio HiFi reads, 37.90 Gb (57.9X) of Illumina PE150 reads, and 94.39 Gb (145.6X) of Hi-C reads for assembly. For RNA, one Iso-seq and one Illumina library were sequenced, from which we obtained 1.58 Gb RNA Iso-seq reads and 9.98 Gb Illumina RNA-seq reads (Table 1).

**Table 1:** Library sequencing data and methods used in this study to assemble the *Mongoloraphidia duomilia* genome.

Sequencing strategy	Clean data (Gb)	Sequencing coverage (x)	Insertion size	Platform	Usage
DNA PacBio HiFi (long-reads)	41.57	64.14	10–20 Kb	PacBio Sequel II	Genome assembly
DNA Illumina (short-reads)	37.90	57.9	350 bp	Illumina NovaSeq 6000	Genome survey
DNA Hi-C	94.39	145.6	350 bp	Illumina NovaSeq 6000	Hi-C assembly
RNA Illumina	9.98	/	350 bp	Illumina NovaSeq 6000	Anno-evidence
RNA ISO-seq	1.58	/	1–10 Kb	PacBio Sequel II	Anno-evidence

### Genome assembly and scaffolding

**Fig. 2:**
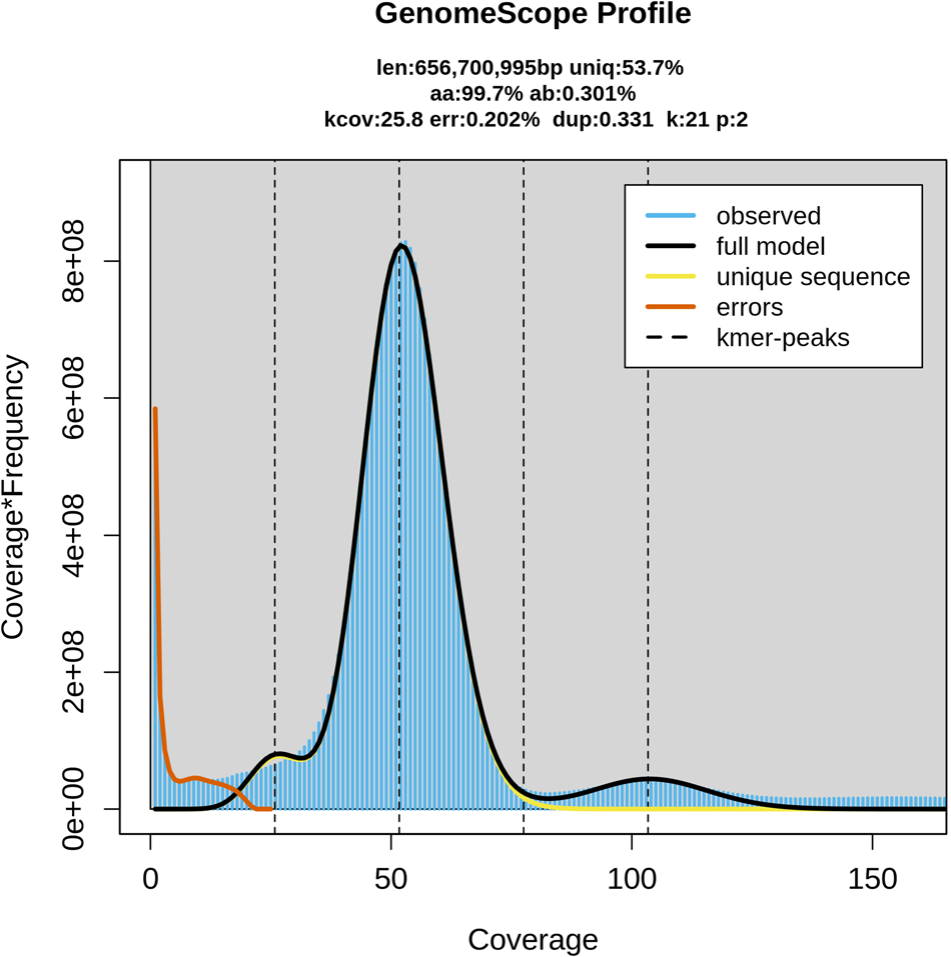
Genome scope profile of the *Mongoloraphidia duomilia* raw reads.

The resulting Illumina sequence data was subjected to quality control and trimming using Fastp version 0.23.0^19^, with the following parameters: quality trimming (>Q20), remove repetitive sequences (-D), trim polyG/X tails (-g -x), proportion of unqualified bases does not exceed 10% (-u 10), reads shorter than 15 bp or with >5 Ns discarded, use overlap regions (overlapping reads), and correct bases (-c). Genome size was estimated using the Illumina short-read sequencing data in the program GenomeScope2 version 2.0^20^ with the k-mer size set as 21 and a maximum k-mer coverage cutoff of 10,000. The genome of *M. duomilia* was predicted to be approximately 656.70 Mb with 46.3% repetitive sequence data and 0.20% heterozygosity (Fig. 2).

**Fig. 3:**
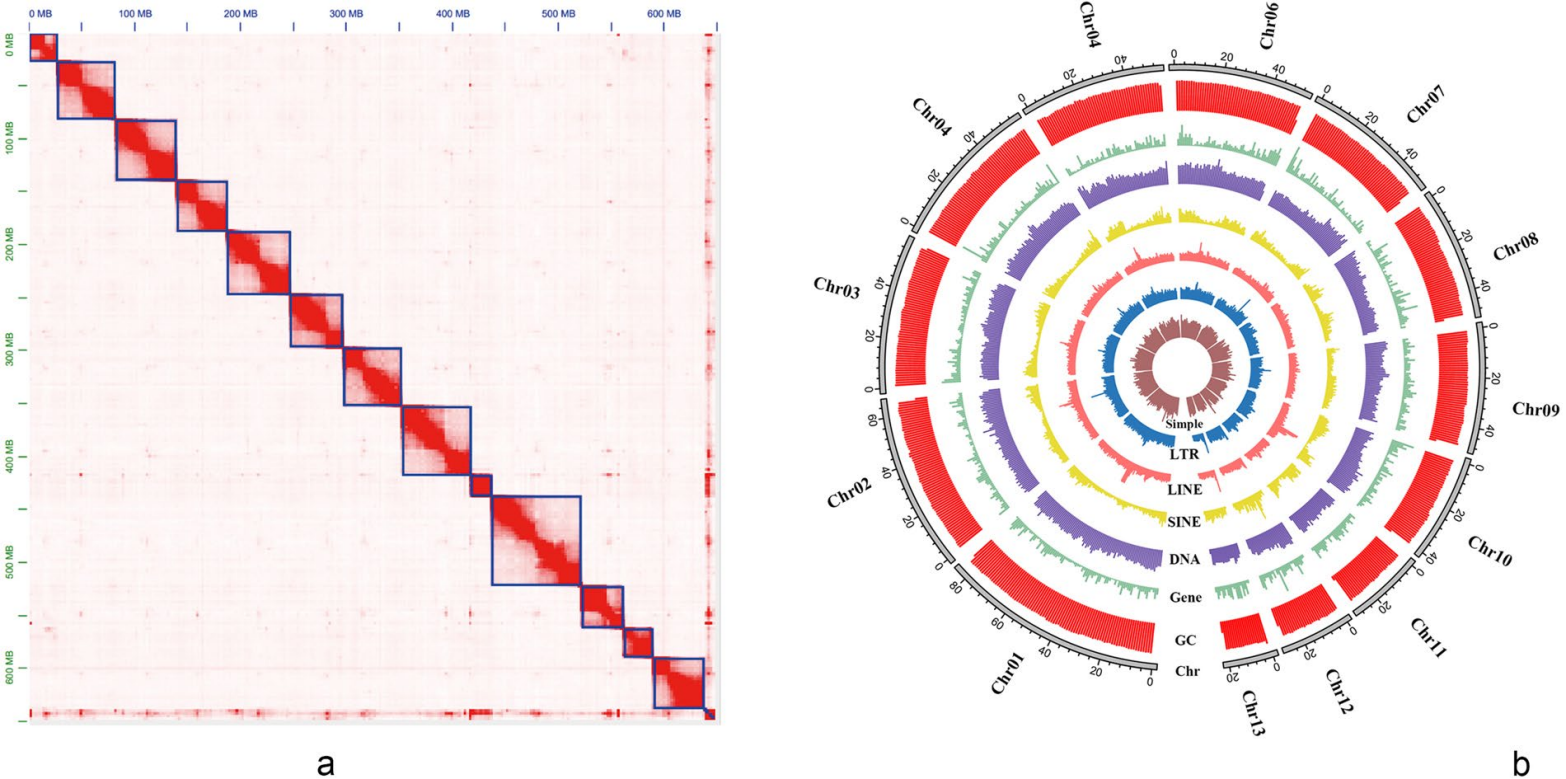
Heatmap of genome-wide Hi-C data and overview of the genomic landscape of *Mongoloraphidia duomilia*. (**a**) The heatmap shows all-by-all interactions among 13 chromosomes of *M. duomilia*. Resolution: 600 kb. There were strong intrachromosomal interactions (blocks on the diagonal line), while interchromosomal interactions were weaker. The frequencies of Hi-C interaction links are represented by the color, which ranges from white (low) to red (high). (**b**) Blocks on the outmost circle represent all 13 chromosomes of *M. duomilia*. Peak plots from outer to inner circles represent the length of each chromosome, the GC content of each chromosome, protein-coding genes, DNA TEs, and the density of repeat sequences (SINE, short interspersed elements; LINE, long interspersed elements; LTR, long terminal repeat elements; simple repeats), respectively.

To assemble the *M. duomilia* nuclear genome, we generated a fasta file from the PacBio HiFi reads bam file using the bam2fasta module in SAMtools version 1.10^21^, and Wtdbg2 version 2.5^22^ was used for preliminary genome assembly, using default parameters. The preliminary assembly was polished with two rounds of Illumina short reads using NextPolish version 1.1.0^23^. Purge_dups version 1.2.6^24^ was used to remove duplications caused by heterozygosity in the assembly. Minimap2 version 2.17-r941^25^ was used for mapping reads during the redundancy removal and short-read polishing steps. Juicer version 1.6.2^26^ and 3D-DNA^27^ were used to anchor primary contigs into chromosomes. Juicebox version 1.11.08^26^ was used to correct errors manually. BUSCO version 5.2.2^28^ with the database insecta_odb10 was used to evaluate the assembly quality. To detect potential contaminant sequences within the assembly, we used the UniVec and NCBI non-redundant preformatted nucleotide blast databases (accessed: 08-Dec-2022) to perform a blastn-like search using MMseqs. 2 version 11^29^ employing the easy-search module with a target sensitivity of 7.5, a maximum e-value threshold of 1e-5, and five hits accepted per query sequence (–s 7.5 –alignment-mode 3 –num-iterations 1 –e 1e-5 –max-accept 5). The resulting chromosome-level genome was 653.56 Mb, comprising 1,467 contigs, with an N50 of 4.60 Mb and the largest contig size of 22.57 Mb, and the GC content of 33.34% (Table 2). The genome assembly is slightly smaller than that of the black-necked snakefly *V. nigricollis* (JAVRKA000000000) (669 Mb) (Wolf *et al*., 2023). Upon Hi-C scaffolding, 97.90% of the assembly was anchored on 13 chromosomes (Table S1), which were well-distinguished from each other based on the chromatin interaction heatmap (Fig. 3a). In total, the length of pseudochromosomes was 639.94 Mb, and ranged from 20.39 Mb to 80.80 Mb in length. The GC content of X chromosome was the lowest (Fig. 3b).

**Table 2:** Genome assembly and scaffolding statistics of *Mongoloraphidia duomilia*.

Characteristics	*M. duomilia*
**Genome assembly**	
Assembly size (Mb)	653.356
Number of scaffolds/contigs	375/1467
Longest scaffold/contig (Mb)	83.804/22.569
N50 scaffold/contig length (Mb)	53.5/4.6
GC	33.34%
Anchored to chromosome (Mb, %)	639.63 (97.90%)
**BUSCO (%)-contigs**	
Complete	97.8
Complete single copy	95.8
Complete duplicated	2
Fragmented	0.6
Missing	1.6
**BUSCO (%)-scaffolds**	
Complete	97.7
Complete single copy	96.3
Complete duplicated	1.4
Fragmented	0.6
Missing	1.7

### Genome annotation

Repetitive elements in the genome of *M. duomilia* were identified by both homology-based and de novo prediction methods. RepeatModeler version 2.0.3^30^ with the LTR discovery pipeline (-LTRStruct) was used to create a de novo repeat library for *M. duomilia*. A custom repeat library was created by combining the de novo repeat library and the Dfam (release 3.8) (http://www.dfam.org/)^31^ and RepBase-20181026 databases (https://www.girinst.org/repbase/)^32^. Repetitive sequences were identified and masked using RepeatMasker version 4.1.2 (http://repeatmasker.org) and the custom library. A total of 366.04 Mb of repeat sequences were identified, constituting 56.01% of the *M. duomilia* genome, including 50.05% interspersed repeats, 0.25% small RNAs, 0.02% satellites, 0.88% simple repeats, and 0.14% complex repeats. Among interspersed repeats, 7.0% of the genome was retroelements, and 7.52% was DNA transposons. Unclassified repeats accounted for 35.63% of the genome (Table S2). The proportion of repetitive elements was higher than *C. pallens* (Neuroptera: Chrysopidae) (206.21 Mb, 38.31%) and *N. ignobilis* (Megaloptera: Corydalidae) (175 Mb, 36.41%) but lower than *V*. *nigricollis* (Raphidioptera: Raphidiidae) (432.20 Mb, 64.6%).

Noncoding RNAs, including transfer RNAs (tRNAs), microRNAs (miRNAs), ribosomal RNAs (rRNAs), and small nuclear RNAs (snRNAs) were also identified in the *M. duomilia* genome. rRNAs, snRNAs, and miRNAs were detected using the Rfam database (release 13.0)^33^ and the program Infernal version 1.1.3^34^. The tRNAs were predicted using tRNAscan–SE version 2.0^35^ with “EukHighConfidenceFilter”. The rRNAs and subunits were predicted using RNAmmer version 1.2^36^. We identified 599 noncoding RNAs (ncRNAs), which contained infrastructural (housekeeping) and regulatory ncRNAs. The numbers of ribosomal RNA (rRNA), transfer RNA (tRNA), small nuclear RNA (snRNA), microRNA (miRNA), long ncRNA (lncRNA), ribozyme, and other ncRNA were 20, 279, 84, 152, 2, 3, and 59, respectively. The rRNAs included 11 5 S rRNAs, eight large subunit rRNAs, and one small subunit rRNA. The tRNAs had 21 isotypes. The snRNAs were classified into 14 snoRNAs (small nucleolar RNA; 9 CD-box, 4 HACA-box) and nine spliceosomal RNAs containing three minor ones. The miRNAs were classified into 67 families, ribozymes into two families, and the other ncRNAs into 11 families (Table S3).

Protein-coding genes were annotated by integrating evidence from (1) *ab initio* prediction, (2) transcriptome-based prediction, and (3) homology-based prediction. For *ab initio* prediction, we used BRAKER version 2.1.5, which automatically trained the predictors Augustus version 3.4.0^37^ and GeneMarkES/ET/EP version 4.68_lic^37^ from transcriptomic data and the OrthoDB10 protein database^38^. Input short transcriptomic alignments were generated with HISAT2 version 2.2.0^39^, and long transcriptomic alignments were produced using Minimap2 version 2.17-r941^25^. For transcriptome-based prediction, the Illumina short-reads (RNA data) were assembled with StringTie version 2.1.4^40^ using the assembled genome as a reference. For homology-based gene prediction, Gene Model Mapper (GeMoMa) annotation pipeline version 1.8^41^ was used to identify *M. duomilia* protein-coding genes based on the protein sequences from *Tribolium castaneum* (Coleoptera), *Bombyx mori* (Lepidoptera), *Drosophila melanogaster* (Diptera), *Apis mellifera* (Hymenoptera), *Chrysopa pallens* (Neuroptera), and *Neoneuromus ignobilis* (Megaloptera), which were downloaded from NCBI. The above results were integrated with MAKER version 3.01.03^42^; genes with a start codon and a stop codon were selected to generate the final gene models. The MAKER pipeline predicted 11,141 PCGs with a mean gene and protein length of 11,334.7 and 571.7 bp, respectively. The numbers of exons and introns per gene were 6.6 and 5.5, respectively, and their corresponding mean lengths were 390.4 and 1657.3 bp.

Gene functional annotation proceeded by searching the UniProtKB (SwissProt + TrEMBL) databases (https://www.uniprot.org/) using Diamond version 2.0.11 (--more -sensitive -e 1e-5)^43,44^. Protein domains and gene ontology (GO) were assigned using eggNOG-mapper version 2.0.1^45^ with the eggNOG version 5.0 database, and using InterProScan version 5.60-92.0^46^ against the databases (Pfam)^47^, Smart (http://smart.embl-heidelberg.de/)^48^, Gene3D version 21.0 (http://gene3d.biochem.ucl.ac.uk/)^49^, Superfamily^50^ and Conserved Domains Database (CDD)^51^. In addition, eggNOG-mapper version 2.0.1 was used to assign Kyoto Encyclopedia of Gene and Genomes (KEGG) pathways as well. Finally, a total of 10,722 (96.68%) genes had a match in the UniProtKB database with at least one record, and 9,968 (89.47%) and 10,679 (95.85%) were predicted to have functional domains by InterProScan and eggNOG, respectively. Genes with 9,233 GO items and 4,329 KEGG pathway terms were identified by combining the InterProScan and eggNOG results. The number of annotated genes in the *M. duomilia* genome was substantially lower than in other annotated neuropterid genomes; for example, 16,200 in *C. pallens*, 14,263 in *N. ignobilis*, and 14,126 in *V. nigricollis*. Notably, our annotation pipeline identified functional domains from InterProScan for most protein-coding genes—89.47% in *M. duomilia* versus 6.9% in *V. nigricollis*.

## Data Records

The raw sequencing data and genome assembly of *M. duomilia* have been deposited at the National Center for Biotechnology Information (NCBI). The PacBio, Illumina, Hi-C, and transcriptome data can be found under identification numbers SRR28813347^52^, SRR28800630^53^, SRR28800631^54^, SRR28800634^55^ and SRR28813348^56^. The assembled genome has been deposited in the NCBI assembly with the accession number JBDIXK010000000^57^. Additionally, the results of annotation for repeated sequences, gene structure, and functional prediction have been deposited in the Figshare database^58^.

## Technical Validation

Two methods were used to evaluate the quality of the genome assembly. Firstly, we assessed assembly completeness using BUSCO v5.2.2 with the reference insect gene set (n = 1,367). 97.8% of the complete BUSCOs were included in the assembled genome (Table 2). Secondly, we calculated the mapping rate as a measure of assembly accuracy. The mapping rates for PacBio, Illumina, short and long RNA reads were 99.95%, 85.71%, 86.53% and 86.72%, respectively. These evaluations collectively reflect the high quality of the genome assembly produced in this study.

### Supplementary information

supplementary tables

## Data Availability

No specific codes or scripts were used in this study. All software used is in the public domain, with parameters clearly described in the Methods section.
